# Obesity trends over 10 years in primary hip and knee arthroplasty—a study of 12,000 patients

**DOI:** 10.1007/s11845-022-03092-w

**Published:** 2022-07-08

**Authors:** Colum Downey, Katie St John, Jeet Chatterji, Adrian Cassar-Gheiti, John M. O’Byrne, Paddy Kenny, James P. Cashman

**Affiliations:** 1 National Orthopaedic Hospital Cappagh, Finglas, Dublin 11, Dublin, Ireland; 2 Connolly Hospital Blanchardstown, Dublin 15, Dublin, Ireland

**Keywords:** Hip and knee arthroplasty, Obesity, Surgery

## Abstract

**Objectives/Aims:**

Obesity and its increasing prevalence are global public health concerns. Following joint replacement, there is evidence to support that obese patients are more likely to suffer complications. We examined 10-year trends in BMI of the primary total hip and total knee replacement cohorts in our institution to discern whether the BMI of these patients has changed over time.

**Methods:**

We examined BMI data of patients who underwent primary hip and knee arthroplasty from our institutional database from January 1, 2010 to December 31, 2019 (***n* = **12,169). We analysed trends in BMI over this period with respect to (i) surgical procedure, (ii) gender, and (iii) age categories.

**Results:**

The overall number of surgical procedures increased over the study period which meant more obese patients underwent surgery over time. Average BMI did not change significantly over time; however, there was a statistically significant increase in BMI in females aged < 45 in both arthroplasty groups.

**Conclusion:**

The average BMI of patients undergoing primary hip and knee arthroplasty in our high-volume tertiary orthopaedic centre has remained relatively unchanged over the past 10 years; however, our local service is caring for a greater number of overweight/obese patients due to the increase in overall volume. This will have significant implications on health care expenditure and infrastructure going forward which further emphasises the importance of ongoing national obesity prevention strategies. The increase in BMI seen in females aged < 45 may mark an impending era of obese younger patients with end-stage osteoarthritis.

**Supplementary information:**

The online version contains supplementary material available at 10.1007/s11845-022-03092-w.

## Introduction

Obesity is a major preventable global health problem which has nearly tripled since 1975 [1]. In 2016, the World Health Organisation (WHO) reported that approximately 1.9 billion adults were overweight (body mass index (BMI) > 25 and < 30 kg/m^2^) and greater than 650 million adults or ~ 13% of the total adult population were obese (BMI > 30 kg/m^2^) [1]. The prevalence of obesity in Europe has recently been estimated at ~ 23% [2]. A recent European health interview survey reported that Ireland had the 2nd highest adult obesity rate in the European Union at 26% [3] and modelling undertaken by the World Health Organisation predicts that by 2030 in Ireland, 47% of both men and women will be obese [4]. Pineda et al. analysed past and present BMI trends across 53 countries in Europe to forecast future trends in obesity and concluded that by 2025, Ireland will have the highest prevalence of obesity in Europe at 43% (95% confidence interval: 28–58%) [5].

Obesity is a risk factor for many diseases including the development and progression of osteoarthritis (OA). Obesity contributes to this process by increasing mechanical stress on the cartilage of weight-bearing joints; however, obesity can also cause osteoarthritis in non-weight-bearing joints. This effect is explained by the systemic inflammatory state that obesity creates mediated by adipose derived cytokines, chemokines, and adipokines. These inflammatory factors negatively impact the integrity of cartilage leading to OA [6].

The most effective treatment for pain and disability associated with end-stage osteoarthritis is total joint arthroplasty. However, evidence in the literature reports increased rates of superficial (OR 1.7–2.2) and deep (OR 2.4) infections following total knee arthroplasty in the obese compared to the non-obese cohort [7]. The evidence in relation to increased risk of complications associated with obesity following total hip arthroplasty is less clear. It has also been shown that the cost of treating obese patients increases as BMI deviates from normal BMI in both total knee and total hip arthroplasty [8–10]. BMI is only one measure of poor physiological state and there has been criticism regarding the use of this measure in isolation to stratify risk categories following arthroplasty surgery for osteoarthritis [11].

In order to identify any trends in BMI over a 10-year period, we performed a retrospective study examining body mass index in those patients who underwent primary total hip or total knee arthroplasty in our high-volume tertiary orthopaedic hospital.

## Methods

In this retrospective cohort study, anonymised data of all patients who had undergone primary total hip or knee arthroplasty in the period of January 1, 2010 to December 31, 2019 in our institution were collected from institutional database (*n* = 12,742). The data was further analysed and those patients for whom no data was available relating to BMI were excluded (*n* = 573/4.5%). The remaining patients (*n* = 12,169) were subcategorised based on procedure, gender, and age. BMI categories were assigned based on the WHO classification depicted in Table 1 below. The BMI data for each group was then statistically analysed using the STRATA system with median BMI and IQR results calculated for each group. Statistical significance was designated to findings with a *p*-value of < 0.05. Ethical approval for the study was granted by our institution’s ethics committee.

**Table 1:** WHO BMI classification

**Nutritional status**	**BMI (kg/m**^**2**^**)**
**Underweight**	< 18.5
**Normal weight**	18.5–24.9
**Pre-obesity**	25–29.9
**Obesity class 1**	30–34.9
**Obesity class 2**	35–39.9
**Obesity class 3**	> 40

## Results

A retrospective review of our institutional database was performed. A summary of the demographics of our study cohorts is summarised separately in Tables 2 and 3.

**Table 2:** Demographic overview of primary total knee arthroplasty cohort

**Primary total knee arthroplasty**											
			**Female**			**Male**			**Total**		
			(*n*)	(%)		(*n*)	(%)		(*n*)	(%)	
**Total**			**3410**	**(61)**		**2177**	**(39)**		**5587**	**(100)**	
**Age**	< 45		48	(1.4)		57	(2.6)		105	(1.9)	
	46–55		310	(9.1)		246	(11.3)		556	(10.0)	
	56–65		962	(28.2)		688	(31.6)		1650	(29.5)	
	66–75		1315	(38.6)		843	(38.7)		2158	(38.6)	
	76–85		731	(21.4)		317	(14.6)		1048	(18.8)	
	< 86		44	(1.3)		26	(1.2)		70	(1.2)	
**BMI (mean)**	18.5–24.9		276	(8.1)		142	(6.5)		418	(7.5)	
	25–29.9		880	(25.8)		697	(32.0)		1577	(28.2)	
	30–34.9		1071	(31.4)		866	(39.8)		1937	(34.6)	
	35–39.9		723	(21.2)		359	(16.5)		1082	(19.4)	
	> 40		460	(13.5)		113	(5.2)		573	(10.3)

**Table 3:** Demographic overview of primary total hip arthroplasty cohort

**Primary total hip arthroplasty**											
			**Female**			**Male**			**Total**		
			(*n*)	(%)		(*n*)	(%)		(*n*)	(%)	
**Total**			**3425**	**(52)**		**3157**	**(48)**		**6582**	**(100)**	
**Age**	< 45		263	(7.7)		306	(9.7)		569	(8.6)	
	46–55		463	(13.5)		524	(16.6)		987	(15.0)	
	56–65		859	(25.1)		913	(28.9)		1772	(26.9)	
	66–75		1054	(30.8)		915	(29.0)		1969	(29.9)	
	76–85		709	(20.7)		460	(14.6)		1169	(17.8)	
	> 86		77	(2.2)		39	(1.2)		116	(1.8)	
**BMI (mean)**	< 18.5		28	(0.8)		0	(0)		28	(0.4)	
	18.5–24.9		771	(22.5)		401	(12.7)		1172	(17.8)	
	25–29.9		1125	(32.9)		1326	(42)		2451	(37.2)	
	30–34.9		894	(26.1)		991	(31.4)		1885	(28.6)	
	35–39.9		425	(12.4)		341	(10.8)		766	(11.6)	
	> 40		182	(5.3)		98	(3.1)		280	(4.3)	

A total of 12,169 (95.5% of all patients) patients were identified as having BMI data available. A total of 5587 of these underwent primary total knee arthroplasty, while 6582 underwent primary total hip arthroplasty.

In the hip arthroplasty group, 52% of patients were female, while 48% were male. In the knee arthroplasty group, 61% of patients were female while 39% were male. The age group 66–75 was the most common age category for both total knee arthroplasty (38.6%) and total hip arthroplasty (29.9%).

**Fig. 1:**
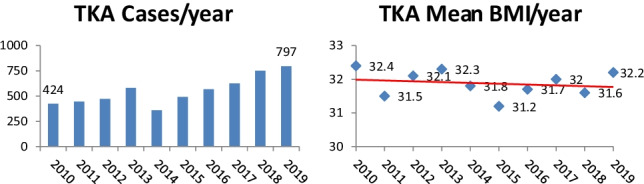
Primary total knee arthroplasty cases and overall mean BMI per year 2010–2019

**Fig. 2:**
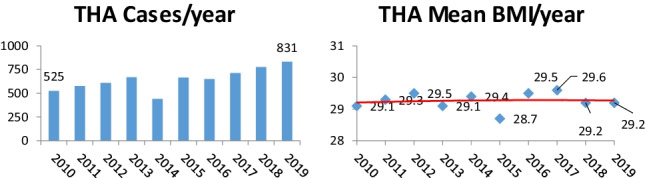
Primary total hip arthroplasty cases and overall mean BMI per year 2010–2019

Analysis revealed that the number of both primary hip and knee arthroplasties performed in our institution increased during the study period (**2010**, 525 and 424; 2019, 831 and 797 respectively (Figs. 1 and 2)). Despite the rise in cases performed, year on year, the median BMI of patients presenting for surgery remained relatively constant (Figs. 1 and 2).

**Fig. 3:**
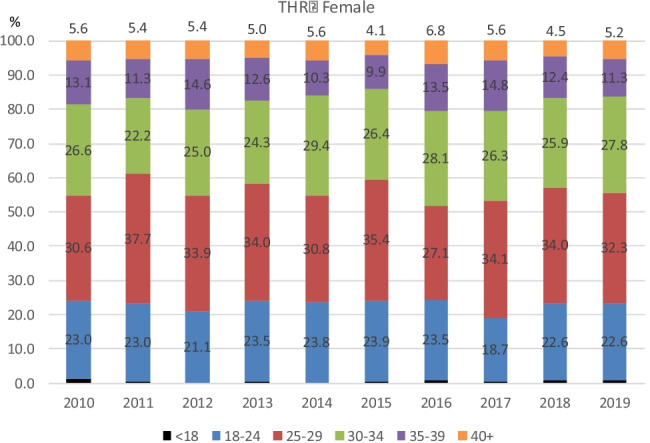
Percentages per BMI class for each year 2010–2019 in females who underwent Primary THR

**Fig. 4:**
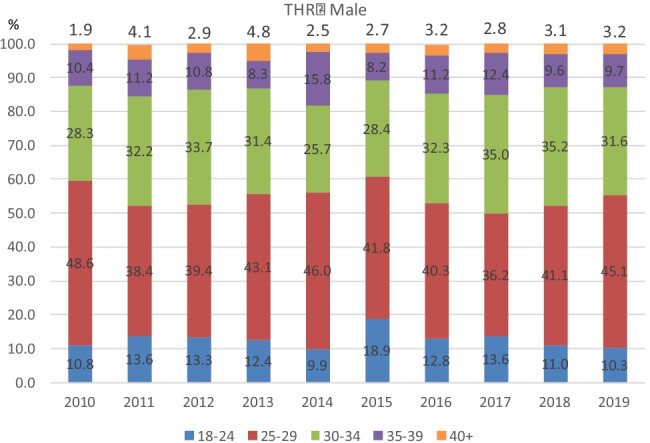
Percentages per BMI class for each year 2010–2019 in males who underwent Primary THR

**Fig. 5:**
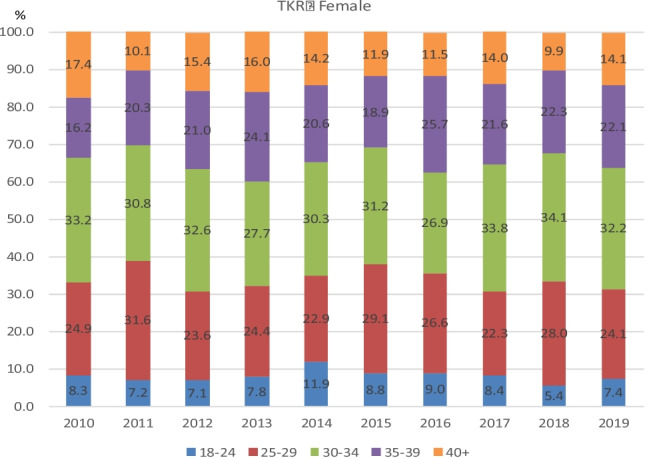
Percentages per BMI class for each year 2010–2019 in females who underwent Primary TKR

**Fig. 6:**
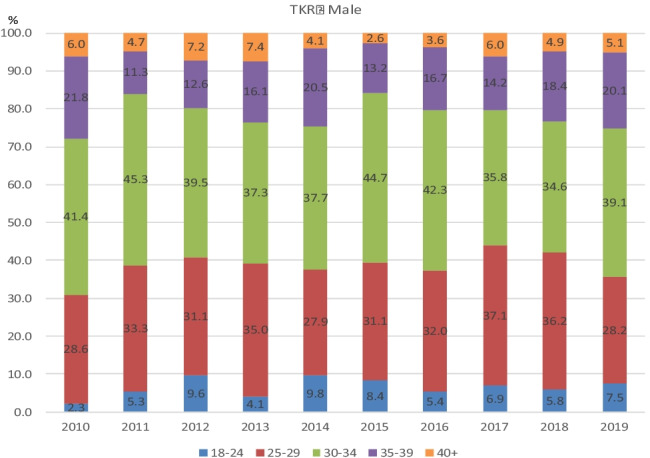
Percentages per BMI class for each year 2010–2019 in males who underwent Primary TKR

Figures 3, 4, 5 and 6 demonstrate the trend in % of patients in each BMI class (normal, overweight, and obese classes 1, 2, and 3) for each year of the study period. As is apparent from the graphs, the % of patients in each BMI class did not vary significantly year on year. The BMI raw data from each group (procedure/sex/age) was statistically analysed and the results were displayed using boxplots (see supplementary data for boxplots for the most common age groups aged 66–75 within each procedure/sex group as examples). These results showed that the trend in median BMI remained relatively constant across both age and gender groups for both hip and knee arthroplasty, with no statistically significant variation found. However, in females aged < 45 years which had undergone total hip or total knee arthroplasty (*p* = 0.03 and *p* = 0.04, respectively), there was a statistically significant increase in BMI.

## Discussion

Taken in concert with the projections of the WHO regarding the global obesity trajectory (1), it is of interest that the BMI of those patients undergoing hip and knee arthroplasty in our institution has remained more or less consistent over the past 10 years. It is worth noting at this point that our country does not have a national policy wherein patients must be below a certain BMI to undergo large joint arthroplasty.

Patients classified as overweight or obese have been designated in the literature as high-risk surgical candidates across many disciplines as they are more at risk of both cardiorespiratory complications and slower/poorer healing—more often due to metabolic disorders [12]. From an orthopaedic perspective, the increased risk of perioperative complications associated with obesity has been described by Abdulla et al. in 2020 in both TKR and THR patients. The authors highlighted that, in this patient group, increase in BMI was associated with increased inpatient medical events and 30-day readmission rates when compared to those patients who were of a normal BMI [13]. Xu et al. have further shown that rates of wound infection also correlated with increases in BMI in the total joint arthroplasty cohort [14].

We examined a large cohort of patients undergoing joint replacement and found that more patients of higher BMIs are now undergoing arthroplasty than 10 years ago. This finding fits with US and UK trends with projections of > 100% growth in rates of both hip and knee primary arthroplasty from 2005 to 2030 [15]. This is significant in terms of projected patient outcomes both functional and psychological and indeed the MDT workload that these patients require. A recent 2020 study by Katakam et al. showed that level 3 obesity (> 40 kg/m^2) in THA increases risk of failure to achieve the 1-year functional outcomes of Hip Disability and Osteoarthritis Outcome Score-Physical Function Short Form Minimal Clinically Important Difference (HOOS-PS and MCID) nearly threefold [16]. To deliver optimal medical care to such patients requires infrastructure beyond what is readily available in our major and regional hospitals at present. Healthcare expenditure will be required on items such as beds, chairs, and radiological equipment as well as staffing if the numbers are to continue to increase as they have in the past decade.

Of note, the percentage of patients in any given year falling into each BMI category remained relatively unchanged over time. Moreover, the percentage of patients falling into each BMI category when subdivided by gender and age also remained relatively unchanged in both hip and knee arthroplasty groups.

While the focus of this study was that of hypothesised uptrends in BMI and surgical implications this may have, a small number of patients in the analysed cohort were found to be underweight, classified as a BMI of < 18. Katakam et al. in 2020 demonstrated that these patients have increased length of stay and 2-year mortality rates post-TKA and THA [17]. Malnourishment alone constitutes a risk factor for OA and it is important to recall that both overweight and underweight patients can be classified as malnourished.

In keeping with international standards, in our large volume arthroplasty unit, patients undergo routine operative pre-assessment by the in-house anaesthetics team prior to formal listing for surgery. At this stage, some patients may have been deemed unsuitable for surgery at this hospital. These patients would have been referred to an acute hospital with high dependency or intensive care supports for their elective surgery. Their BMI results would thus not have been included in our data. Of note, since 2016 the local anaesthetic pre-assessment team have been collecting annual figures on those patients who were deemed unfit for surgery based on BMI at pre-assessment. This figure has been low and stable at 1.3–1.5% annually and without any significant trend between 2016 and 2019.

**Fig. 7:**
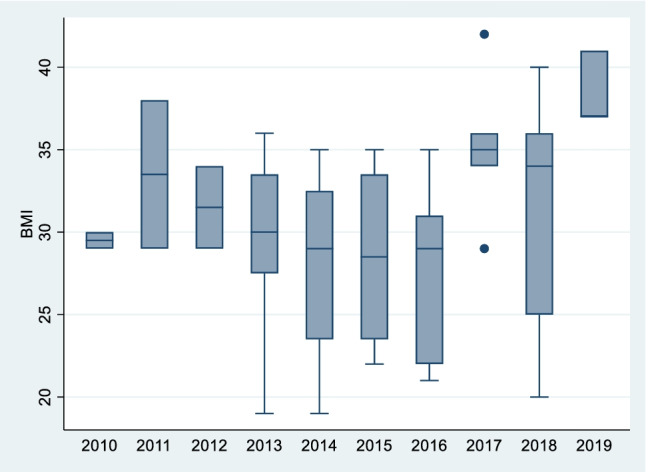
Female TKR < 45 BMI trend

**Fig. 8:**
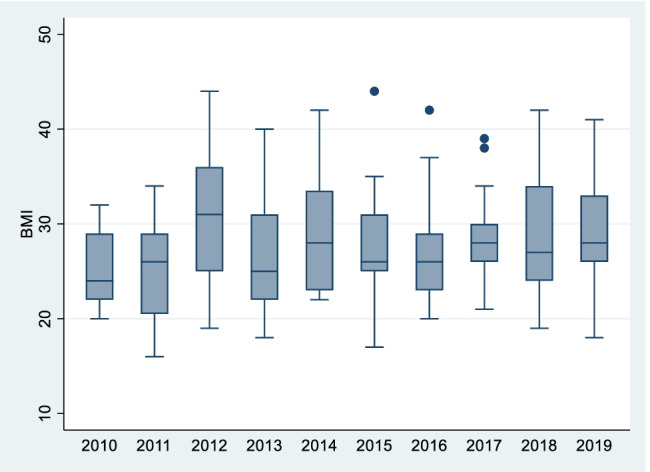
Female THR < 45 BMI trend

In terms of the trend in BMI over the 10-year period, the only statistically significant upward trend seen in both the TKR and THR groups was in women aged < 45 (*p* = 0.04 and *p* = 0.03, respectively) (see Figs. 7 and 8). The median BMI for TKRs in this group in 2010 was 29.5 (29 to 30) and in 2019, 37 (37 to 41). In THRs for the same group, the median BMI in 2010 was 24 (22 to 29), while in 2019 this figure had increased to 28 (26 to 33).

These findings reflect the results of a recent study by Reynolds et al. who looked at BMI trends in pregnant women in Ireland between 2010 and 2017 (*n* = 67,949) [18]. Similarly, the authors found rates of obesity among patients increased throughout the 7-year period, with the largest increase in BMI seen in the 20–24 age bracket (24 to 25.4 kg/m^2) followed by those in the 25–29 age group.

Although the females aged < 45 years group which underwent arthroplasty represents only 48 patients out of the total in our study, the concern remains that these findings may mark an impending era of young patients requiring arthroplasty for end-stage osteoarthritis. We may see an eventual merging of the growing paediatric obesity pandemic to meet the earlier adult onset of obesity. These patients may require multiple revision arthroplasties and may endure the risk of the corresponding complications that go with same. This would have obvious implications in terms of healthcare expenditure and burdening the already long waiting lists of elective arthroplasty practice for years to come.

From an ethical perspective it is worth mentioning that in the UK in 2018, a number of NHS clinical commissioning groups suggested the rationing of surgeries in patients who smoke or are obese, meaning patients would have to quit smoking or lose weight prior to being considered a surgical candidate for a number of procedures [19]. This caused considerable controversy given the idea suggests that personal responsibility is the key reason to ration surgical care. Such practices, while they are likely to cut down public healthcare expenditure, may not have the desired impact in terms of fostering positive health behaviours among the targeted patient group, given that the patients do not engage the true cycle of change having made a choice to do so, but have been forced. Roth et al. in 2020 examined whether introducing BMI cutoffs for patients being considered for TKA would be beneficial in terms of post op complications/mortality and readmission rates [20]. The study found that such measures of designating illegibility for surgery may deny a large number of patients an uneventful post op course and excellent results in terms of functional outcomes and pain relief.

## Conclusion

While trends in mean BMI among primary hip and knee arthroplasty recipients have remained relatively stable over the past decade in our large volume arthroplasty unit, we should not be falsely reassured by this data. The overall numbers of overweight and obese patients requiring total hip and knee arthroplasty have risen over our 10-year study period as overall volume has increased. This presents a challenge in terms of optimally delivering an orthopaedic service to a subset of patients which (i) have an increased risk of complications and (ii) whom require investment in size-appropriate infrastructure which both carry significant financial implications. Obesity is complex and prevention and treatment are key. We welcome the recent local publication of the Model of Care for the Management of Overweight and Obesity 2021 [21] and look forward to the results of the proposed implementation of phases 1–3 (2021–2031) over the next 10 years in our country.

## Supplementary Information

Below is the link to the electronic supplementary material.

Supplementary file1 (DOCX 39 KB)
