# Supplementary material for: Obesity trends over 10 years in primary hip and knee arthroplasty—a study of 12,000 patients
Source: Ir J Med Sci. 2022 Jul 8;192(3):1197–203. doi: 10.1007/s11845-022-03092-w (PMC10250270; doi:10.1007/s11845-022-03092-w)
Supplement: Supplementary file 1 — Supplementary file1 (DOCX 39 KB) [file 11845_2022_3092_MOESM1_ESM.docx]

**Supplementary Data (Figures A – D).**

**Figure A.** Boxplot data summary for those females aged 66-75 years who underwent Primary THR

**Figure B.** Boxplot data summary for those males aged 66-75 years who underwent Primary THR

**Figure C.** Boxplot data summary for those females aged 66-75 years who underwent Primary TKR

**Figure D.** Boxplot data summary for those males aged 66-75 years who underwent Primary TKR
